# Characterization of Emerging Human *Dirofilaria repens* Infections, Estonia, 2023

**DOI:** 10.3201/eid3109.241890

**Published:** 2025-09

**Authors:** Kalev Nõupuu, Maare Mõtsküla, Riina Pulges, Mikk Pauklin, Urmas Saarma

**Affiliations:** Tartu University Hospital, Tartu, Estonia (K. Nõupuu, R. Pulges, M. Pauklin); Estonian University of Life Sciences, Tartu (M. Mõtsküla); University of Tartu, Tartu (U. Saarma).

**Keywords:** dirofilariasis, emerging disease, parasites, mosquitoes, vector-borne infections, mosquito-borne, zoonoses, Estonia

## Abstract

Mosquitoborne diseases are a growing threat to public health worldwide. Human dirofilariasis, caused by the nematode *Dirofilaria repens* and transmitted by mosquitoes from various genera, has recently expanded into new areas of Europe. In this article, we report molecularly confirmed autochthonous human *D. repens* infections in Estonia.

Human dirofilariasis, caused by nematodes of the genus *Dirofilaria*, is a mosquitoborne parasitosis with growing public health importance. In Europe, the main causative species is *D. repens*, and infections with *D. immitis* are less frequent. Mosquitoes play a crucial role in the transmission of infectious larvae, and suitable species span various mosquito genera, including *Aedes*, *Anopheles*, and *Culex* (*1*). The definitive hosts of *D. repens* nematodes are domestic and wild carnivores. Humans are considered accidental hosts, in whom the parasitic larvae typically develop into a nonfertile stage. Although most human cases are subclinical, *D. repens* infection might occasionally result in subcutaneous swelling with subsequent development of subcutaneous nodular lesions. The clinical manifestations can include a mobile mass within the conjunctiva (*2*) and can lead to irreversible ocular damage (*3*). In rare cases, microfilaremia has been described (*3*,*4*). The distribution of *D. repens* nematodes includes countries in Europe, Africa, Middle East, Asia, and South America (*1*). In Europe, the parasite has recently spread north, and cases of human dirofilariasis caused by *D. repens* infection have emerged in Lithuania, Latvia, and Finland (*5*,*6*). *D. repens* nematodes have been reported in dogs in Estonia (*7*,*8*). Here, we describe 2 molecularly confirmed human *D. repens* infection cases from Estonia.

In February 2023, a 46-year-old woman in Estonia with no travel history abroad was referred to a neurology service for investigation of recurrent headache and painful facial subcutaneous nodular lesions with accompanying edema. The patient described a 1.5–2 cm palpable lesion that remained at the same location for 1–2 days before disappearing and reappearing elsewhere. Her symptoms persisted for 1 month, during which she observed nodules and edema on her upper (Figure 1, panel A) and lower eyelids, forehead, lips, and scalp. Two months later, a permanent 1.5 cm subcutaneous nodule developed in her right upper eyelid (Figure 1, panel B), and the patient was referred to an ophthalmologist. We surgically removed the nodule and sent it for histopathologic examination. The parasite in the nodule was confirmed as *Dirofilaria* spp. (Figure 1, panel C). After the removal of the parasite, her symptoms resolved and did not recur.

**Figure 1:**
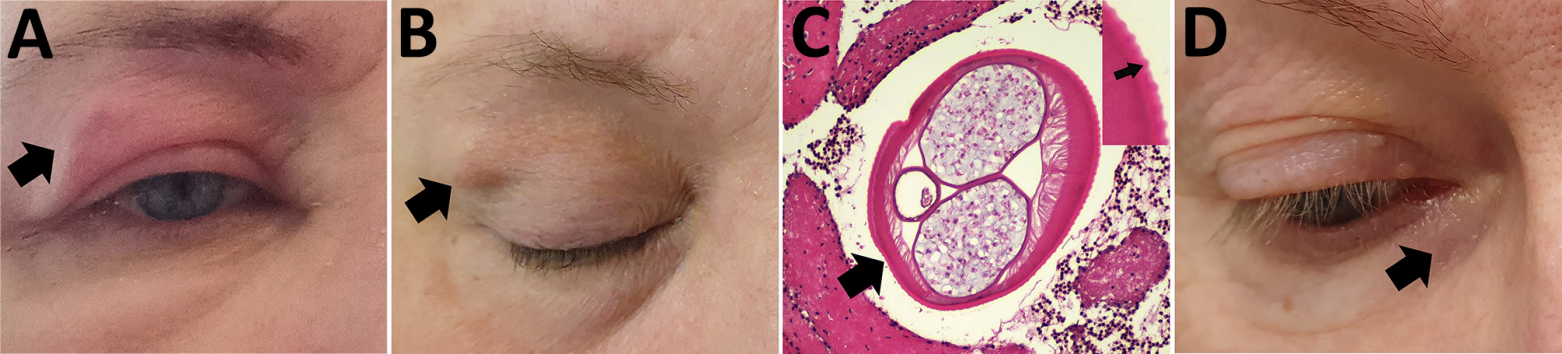
Autochthonous *Dirofilaria repens* infections in 2 women, Estonia, 2023. A–C) Case-patient 1, showing a painful subcutaneous lesion with edema on her right eyelid (A; black arrow). Two months later, the edema resolved, but a 1.5 cm subcutaneous nodule persisted (B; black arrow). Histopathologic examination of the removed nodule confirmed *Dirofilaria* spp. (C; larger black arrow); distinctive transverse striations on the cuticle with average distance of 12 µm between the peaks suggested *D. repens* (inset; small black arrow). Hematoxylin and eosin stain; original magnification ×100. The diameter of the parasite was 390 × 529 µm. D) Case-patient 2, showing a subcutaneous nodule under the right lower eyelid (black arrow).

In February 2023, a 77-year-old woman in Estonia with no travel history abroad was referred to an ophthalmologist because of a nodular lesion under the right lower eyelid (Figure 1, panel D). The patient reported an episode of a mild, painful swelling that preceded the formation of the lesion. The oval lesion (1 × 1.5 cm) was located nasally under the right lower eyelid next to the orbital rim, was not painful and was freely movable, and was not adhered to the orbital rim or lacrimal sac. Initially, we recommended conservative observation; however, over the next 5 months, the lesion grew, and mild edema reappeared. Because of a clinical suspicion of a tumor, we surgically excised the lesion. The specimen was submitted for a histopathologic examination, which identified *Dirofilaria* spp.

No treatment guidelines for human *D. repens* infection have been established; however, surgical removal of the parasite is usually sufficient, and pharmacologic treatment is rarely necessary (*3*). After the removal of the encapsulated parasite in the 2 cases described, the symptoms resolved. No systemic treatment was applied. Additional data for both human cases are provided (Appendix).

We conducted molecular genetic analysis for species identification and phylogenetic inference on samples collected from both patients (Appendix). We submitted sequences to GenBank (accession nos. PQ608665, PQ608666, and PQ608671). We conducted species identification on the basis of the 331 bp sequence by using homology search with nucleotide BLAST (https://blast.ncbi.nlm.nih.gov) and identified both pathogens as *D. repens* nematodes. On the basis of the longer fragment of *cox1* (570 bp), we built a phylogenetic network that comprised the human isolate *In2* from Estonia and 38 highly homologous sequences from GenBank (Figure 2). In that network, the human isolate *In2* from Estonia formed a unique haplotype 1, suggesting low-level divergence from the central haplotype 2. Of note, *D. repens* is genetically closest to a newly described species, *D. asiatica* (*9*) (Appendix).

**Figure 2:**
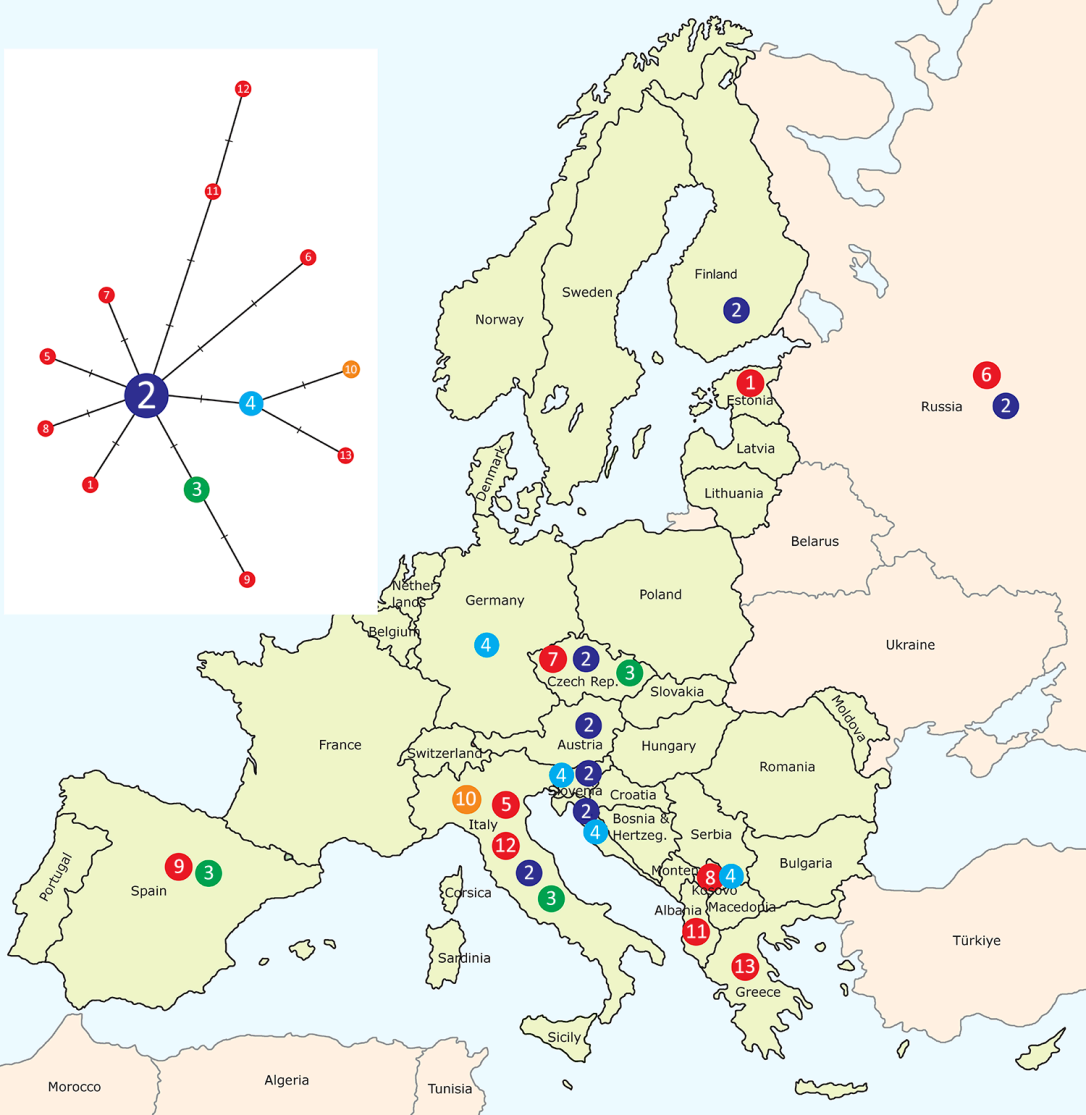
Distribution map of emerging *Dirofilaria repens* haplotypes (n = 13) in Europe, including samples from Estonia, 2023. Haplotypes were derived from the median joining network analysis (left), illustrating relationships of *D. repens* isolates (n = 39) from Estonia and other countries. We established relationships on the basis of partial mtDNA *cox1* gene sequence variation (570 bp). The circled number is the haplotype identification number. Red circles represent haplotypes with unique sequences. Haplotypes represented by >2 are in different colors. Each crossed bar equates to a single-nucleotide polymorphism differentiating the haplotypes. Of note, the human isolate *In2* from Estonia has a unique haplotype 1, which is closely related to the central haplotype 2, reported previously in *D. repens* isolates from humans, dogs, and mosquitoes in various countries (Appendix Table 2). mtDNA, mitochondrial DNA.

We report 2 autochthonous human *D. repens* infections in Estonia, highlighting the importance of recognizing this emerging threat. The geographic distribution of *D. repens* may have expanded because of climate change, enabling the parasite and its vectors to adapt in colder regions and spread the infection northward (*3*,*8*). Therefore, it is necessary to increase the awareness of the parasite among healthcare professionals working in Estonia. In both of these cases, the diagnosis was delayed, and several unnecessary tests were conducted because of a lack of knowledge about dirofilariasis. The common nodular lesions in the facial region might mimic tumors, granulomas, or cysts (*10*). Because no commercially available tests are available to diagnose *D. repens* infection from blood samples, molecular analysis of the parasite is essential for diagnosis (*3*).

Appendix Additional information about characterization of emerging human *Dirofilaria repens* infections, Estonia, 2023.
