# Supplementary material for: Characterization of Emerging Human Dirofilaria repens Infections, Estonia, 2023
Source: Emerg Infect Dis. 2025 Sep;31(9):1854–7. doi: 10.3201/eid3109.241890 (PMC12407190; doi:10.3201/eid3109.241890)
Supplement: Appendix — Additional information about characterization of emerging human Dirofilaria repens infections, Estonia, 2023. [file 24-1890-Techapp-s1.pdf]

Article DOI: https://doi.org/10.3201/eid3109.241890

EID cannot ensure accessibility for supplementary materials supplied by authors. Readers who have difficulty accessing supplementary content should contact the authors for assistance.

Characterization of Emerging Human *Dirofilaria repens* Infections, Estonia, 2023

Appendix

Additional data for human cases

Case 1. The patient (a woman, 46 years of age), had a medical history of clinical depression and allergic rhinitis, for which sertraline and cetirizine were administered regularly. The complete blood count was normal, with no elevation of the eosinophil level. The only abnormality was an elevated immunoglobulin E (IgE) level at 119 kU/L (normal <87 kU/L). Initial diagnostic hypothesis included angioedema, prompting treatment with antihistamines and corticosteroids, but these interventions proved ineffective. The patient was referred to an allergologist who suspected that the edema could be related to sertraline use, as periorbital edema and angioedema are known side effects of this medication. Based on the clinical data, *D. repens* was suspected as a causative agent. The patient was referred to an infectious disease specialist for further examination. Serologic and coprological tests for other parasitic diseases were negative. No systemic treatment was prescribed, and the patient was followed. After the removal of the parasite, all the symptoms disappeared and did not recur. At the 1-year follow-up visit, the IgE level had dropped to 43 kU/L.

Case 2. The patient (a woman, 77 years of age), was referred to an infectious disease specialist. The complete blood count was normal, with no elevation of the eosinophil level. The only abnormality was the elevation of IgE value (137 kU/L; normal <87 kU/L). The computed tomography (CT) scans of the lungs and abdomen did not reveal any abnormalities. The patient was monitored, and a follow-up blood testing conducted 6 months later revealed a decrease in the IgE level to 94 kU/L. After the removal of the encapsulated parasite, the symptoms disappeared and the IgE levels decreased. No systemic treatment was applied.

It is worth noting that both cases had no travel history abroad. In Estonia, there is exposure to mosquitoes everywhere, even in the cities (*1*). Therefore, the patients had exposure to local mosquito bites.

Parasite isolation, DNA extraction, PCR, and sequencing of the mtDNA cox1 gene

Subcutaneous nodules of parasites, surgically removed from two human patients, were sent to histopathology service. The nodules were fixed immediately in formalin and inserted into paraffin blocks. Paraffin slices were stained with hematoxylin and eosin. The diagnosis given by the histopathology service was *Dirofilaria* sp. DNA was extracted from paraffin-embedded samples (about 1-year-old), using the *AllPrep DNA/RNA FFPE Kit* (Qiagen, Hilden, Germany) according to the manufacturer's protocols. PCR amplification of the mitochondrial DNA cytochrome c oxidase subunit I (*cox1*) gene fragments was carried out with two newly developed primer pairs. For species identification, primers Dir1F and Dir2R were used, yielding a 333 bp PCR product. For phylogenetic analysis, primers Dir1F and Dir1R were used, yielding a 684 bp fragment (Appendix Table 1).

Appendix Table 1. Primers designed for PCR amplification of *Dirofilaria repens* mtDNA *cox1* gene fragments. The primer pair Dir1F-Dir2R, with PCR product size of 333 bp, was used for species identification, whereas primers Dir1F-Dir1R (684 bp) for phylogenetic analysis

Primer names	Primer sequences	Nucleotide positions*	PCR product size
Dir1F	GATTGGTGGTTTTGGTAATTGGATG	2516–2540	684 bp
Dir1R	GTACGAGTATCAATATCCAAACCAG	3175–3199	
Dir1F	GATTGGTGGTTTTGGTAATTGGATG	2516–2540	333 bp
Dir2R	CAAACAAACATACTAATCTGATCTAAAGT	2820–2848	

*According to *D. repens* sequence KX265049 in the GenBank database.

The PCR reactions were carried out in a volume of 20 µL, using *HOT FIREPol MultiPlex Mix* (Solis BioDyne, Tartu, Estonia), 0.25 µM of each primer and 10–50 ng of template DNA. Touchdown PCR cycling conditions were: 95°C for 12 min, followed by 10 cycles of 95°C for 20 s, 55°C for 30 s (with annealing temperature reduced in each cycle by 0.5°C) and 72°C for 45 s; followed by 27 cycles of 95°C for 20 s, 50°C for 30 s, 72°C for 45 s; and finished with a final elongation step at 72°C for 2 min. The PCR products were divided into two, of which 10 µL were examined on a 1% agarose gel electrophoresis in 1x TAE buffer. The remaining 10 µL was used for PCR product purification, for which a mixture of FastAP alkaline phosphatase and Exonuclease I (Thermo Scientific, Waltham, USA) was added, 1 unit of each. The mixture was incubated at 37°C for 30 min and enzymes were inactivated at 80°C for 15 min. Sequencing was performed at the Core Facility of Genomics (Institute of Genomics, University of Tartu, Estonia). Both DNA strands were sequenced, using the same primers as for the initial PCR. PCR and sequencing with primers Dir1F-Dir2R were successful for both isolates and produced

identical sequences with a final length of 331 bp (corresponding to positions 2517–2847 in the full mitogenome sequence of *D. repens* from the GenBank database, accession code KX265049) (*2*). It is noteworthy that these new primer pairs produced strong and specific PCR products, suggesting that the primer pair yielding the shorter fragment can be useful for the analysis of degraded samples. However, primers Dir1F-Dir1R that yielded a longer PCR product, the PCR failed for isolate In1 and was successful only for isolate In2: the resultant sequence was 683 bp in length (corresponding to positions 2517–3199 in KX265049). The 331 bp *cox1* sequences of both isolates, as well as the 683 bp sequence of isolate In2, were submitted to GenBank and can be found under the following accession codes: PQ608665 (haplotype In1, 331 bp), PQ608666 (In2, 331 bp) and PQ608671 (In2, 683 bp).

Sequence assembly, species identification and phylogenetic analysis

Consensus sequences were assembled in the program Codon Code Aligner v.11.0.1 (https://www.codoncode.com/index.htm; CodonCode Corporation, Massachusetts, USA) and the quality was assessed by eye-checking the chromatograms. Species identification was performed by database homology search, using the publicly available program Nucleotide BLAST (nBLAST) (https://blast.ncbi.nlm.nih.gov). Based on the 331 bp sequence of *cox1*, the search with nBLAST retrieved 88 sequences of *D. repens* from various countries with 100% sequence identity. Thus, the species was identified as *D. repens*. nBLAST homology search with the longer 683 bp sequence of *cox1* revealed no identical sequence, the closest sequences had at least one nucleotide difference. Sequences with homology >97% in relation to the In2 sequence were retrieved from GenBank and aligned in Bioedit v.7.7.1 (*3*). As a result, a dataset of 39 sequences and 570 bp in length was formed. Based on this, a median-joining network was calculated using the program Network v.10.2.0.0 (http://www.fluxus-engineering.com, Fluxus Technology Ltd, 2004) (*4*), with both indels and point mutations considered. A star-like network was inferred, where the Estonian isolate In2 formed a unique haplotype 1 (Figure 2 in main text), positioning just a single nucleotide difference apart from the central haplotype 2, which comprised *D. repens* isolates from many countries and different host species.

D. repens can be confused with a newly described species *D. asiatica* (formerly *D. sp. hongkongensis*), which is genetically closest to *D. repens* (*5*). Based on the aligned 570 bp sequences of *cox1*, a neighbor-joining (NJ) tree was constructed, using MEGA11 (*6*), with 1000 bootstrap pseudoreplicates. The analysis included the *D. repens* isolate from Estonia (PQ608671)

together with the 38 highly homologous sequences of *D. repens* from the GenBank database, plus *D. asiatica* and *D. immitis* as outgroup taxa (Appendix Table 2). The NJ analysis confirmed that *D. asiatica* is a sister taxon to *D. repens*, whereas *D. immitis* was more distant (Appendix Figure). The Estonian haplotype 1 was closest to haplotypes 3, 7 and 9.

Appendix Table 2. Mitochondrial DNA *cox1* (570 bp) haplotypes of *Dirofilaria repens* isolates, used to construct the phylogenetic network (Figure 2 in main text) and neighbor-joining tree (Appendix Figure). Note that the outgroup sequences of *D. asiatica* and *D. immitis* were used only for constructing the neighbor-joining tree (Appendix Figure)

Species	Host	Country code, ISO 3166–1 α-3	GenBank accession code	Haplotype no. (cox1; 570 bp) (colors are according to Figure 2 in main text)	Reference
D. repens	Human	EST	PQ608671	1	This study
D. repens	Dog	CZE	MW675691	2	(*7*)
D. repens	Dog	CZE	MW675692	3	(*7*)
D. repens	Dog	CZE	MW675693	4	(*7*)
D. repens	Human	HRV	KX265049	2	(*2*)
D. repens	?	ITA	KX265047	5	(*2*)
D. repens	Dog	ITA	KX265048	4	(*2*)
D. repens	Human	CZE	KR998257	2	(*8*)
D. repens	Dog	RUS	MN200335	2	(*9*)
D. repens	Dog	RUS	MN200336	6	(*9*)
D. repens	Human	ITA	PP465883	2	(*10*)
D. repens	*Anopheles sp.*	SVN	PQ219594	2	(*11*)
D. repens	Dog	ITA	MT345575	2	(*12*)
D. repens	Human	HRV	MT847642	4	(*13*)
D. repens	Human	CZE	KR998259	7	(*8*)
D. repens	Dog	AUT	MW590257	2	(*14*)
D. repens	*Anopheles plumbeus*	AUT	MF695085	2	(*15*)
D. repens	*Coquillettidia richiardii*	SVN	PQ219575	4	(*11*)
D. repens	*Anopheles daciae*	DEU	KF692102	4	(*16*)
D. repens	Human	CZE	MW017212	2	(*17*)
D. repens	Human	SVN	OP494269	2	(*18*)
D. repens	Dog	FIN	KY828979	2	(*19*)
D. repens	Human	FIN	KY828978	2	(*19*)
D. repens	Human	SVN	OP494268	2	(*20*)
D. repens	Dog	SVN	OP494253	2	(*21*)
D. repens	Dog	SVN	OP494252	2	(*21*)
D. repens	Dog	SVN	OP494254	2	(*21*)
D. repens	Dog	XKX	PP552047	4	(*22*)
D. repens	Dog	XKX	PP552045	8	(*22*)
D. repens	Human	ESP	MH780816	3	(*23*)
D. repens	Human	ESP	MH780817	9	(*23*)
D. repens	Human	ITA	MT683122	10	(*24*)
D. repens	Human	ITA	MT683121	10	(*24*)
D. repens	Human	ALB	JF461458	11	(*25*)
D. repens	Wolf	ITA	DQ358814	3	(*26*)
D. repens	Dog	CPV	KT901783	3	(*27*)
D. repens	Dog	ITA	AJ271614	12	(*28*)
D. repens	Human	GRC	MK210632	13	(*29*)
D. repens	Human	ITA	OP024281	3	(*30*)
D. asiatica	Dog	LKA	PV523842	Outgroup	(*5*)
D. immitis	*Leopardus pardalis*	BRA	OR434081	Outgroup	(*31*)

References

1. Kirik H, Tummeleht L, Kurina O. Rediscovering the mosquito fauna (*Diptera*: *Culicidae*) of Estonia: an annotated checklist with distribution maps and DNA evidence. Zootaxa. 2022;5094:261–87. PubMed https://doi.org/10.11646/zootaxa.5094.2.3

2. Yilmaz E, Fritzenwanker M, Pantchev N, Lendner M, Wongkamchai S, Otranto D, et al. The mitochondrial genomes of the zoonotic canine filarial parasites *Dirofilaria* (*Nochtiella*) *repens* and *Candidatus Dirofilaria* (*Nochtiella*) *hongkongensis* provide evidence for presence of cryptic species. PLoS Negl Trop Dis. 2016;10:e0005028. PubMed https://doi.org/10.1371/journal.pntd.0005028

3. Hall T. BioEdit: a user-friendly biological sequence alignment editor and analysis program for Windows 95/98/NT. Nucleic Acids Symp Ser. 1999;41:95–8.

4. Bandelt HJ, Forster P, Röhl A. Median-joining networks for inferring intraspecific phylogenies. Mol Biol Evol. 1999;16:37–48. PubMed https://doi.org/10.1093/oxfordjournals.molbev.a026036

5. Colella V, Young ND, Manzanell R, Atapattu U, Sumanam SB, Huggins LG, et al. *Dirofilaria asiatica* sp. nov. (*Spirurida*: *Onchocercidae*) - Defined using a combined morphological-molecular approach. Int J Parasitol. 2025;55:461–74. PubMed https://doi.org/10.1016/j.ijpara.2025.04.006

6. Tamura K, Stecher G, Kumar S. MEGA11: molecular evolutionary genetics analysis version 11. Mol Biol Evol. 2021;38:3022–7. PubMed https://doi.org/10.1093/molbev/msab120

7. Jurankova J, Senkyrikova Mitkova B, Novotna M, Hofmannova L, Cervena B, Bowman DD, et al. Further data on the distribution of *Dirofilaria* spp. in the Czech Republic in dogs. Folia Parasitol (Praha). 2022;69:2022.007

8. Matějů J, Chanová M, Modrý D, Mitková B, Hrazdilová K, Žampachová V, et al. *Dirofilaria repens*: emergence of autochthonous human infections in the Czech Republic (case reports). BMC Infect Dis. 2016;16:171. PubMed https://doi.org/10.1186/s12879-016-1505-3

9. Shaikevich E, Bogacheva A, Ganushkina L. *Dirofilaria* and *Wolbachia* in mosquitoes (*Diptera*: *Culicidae*) in central European Russia and on the Black Sea coast. Parasite. 2019;26:2. PubMed https://doi.org/10.1051/parasite/2019002

10. Mus E, Viani A, Domenis L, Maradei F, Valastro A, Marucci G, et al. First case of human ocular dirofilariasis in the Aosta Valley Region: clinical management and morphological-molecular confirmation. Pathogens. 2025;14:423. PubMed https://doi.org/10.3390/pathogens14050423

11. Glinsek Biskup U, Knap N, Adam K, Knapic T, Zakotnik S. *Dirofilaria repens* isolate 22-207 cytochrome c oxidase subunit I (COX1) gene, partial cds; mitochondrial. 2024 [cited 2025 May 8]. https://www.ncbi.nlm.nih.gov/nuccore/PQ219594

12. Barlozzari G, Felice T, Salvato L, Conti R, De Liberato C, Furzi F, et al. Usual or unusual presentations of *Dirofilaria repens* in two sibling dogs: a case report. Parasitol Res. 2021;120:109–15. PubMed https://doi.org/10.1007/s00436-020-06926-7

13. Pupić-Bakrač A, Pupić-Bakrač J, Beck A, Jurković D, Polkinghorne A, Beck R. *Dirofilaria repens* microfilaremia in humans: case description and literature review. One Health. 2021;13:100306. PubMed https://doi.org/10.1016/j.onehlt.2021.100306

14. Sonnberger BW, Graf B, Straubinger RK, Rackl D, Obwaller AG, Peschke R, et al. Vector-borne pathogens in clinically healthy military working dogs in eastern Austria. Parasitol Int. 2021;84:102410. PubMed https://doi.org/10.1016/j.parint.2021.102410

15. Übleis SS, Cuk C, Nawratil M, Butter J, Schoener E, Obwaller AG, et al. Xenomonitoring of mosquitoes (*Diptera*: *Culicidae*) for the presence of filarioid helminths in Eastern Austria. Can J Infect Dis Med Microbiol. 2018;2018:9754695. PubMed https://doi.org/10.1155/2018/9754695

16. Kronefeld M, Kampen H, Sassnau R, Werner D. Molecular detection of *Dirofilaria immitis, Dirofilaria repens* and *Setaria tundra* in mosquitoes from Germany. Parasit Vectors. 2014;7:30. PubMed https://doi.org/10.1186/1756-3305-7-30

17. Gebauer J, Ondruš J, Kulich P, Novotný L, Sałamatin R, Husa P, et al. The first case of periorbital human dirofilariasis in the Czech Republic. Parasitol Res. 2021;120:739–42. PubMed https://doi.org/10.1007/s00436-020-07003-9

18. Kramar U, Soba B, Cvetko A, Cvetko B. *Dirofilaria repens* isolate SI21-1369 cytochrome c oxidase subunit I (COX1) gene, partial cds; mitochondrial. 2022 [cited 2025 May 8]. https://www.ncbi.nlm.nih.gov/nuccore/OP494269

19. Pietikäinen R, Nordling S, Jokiranta S, Saari S, Heikkinen P, Gardiner C, et al. *Dirofilaria repens* transmission in southeastern Finland. Parasit Vectors. 2017;10:561. PubMed https://doi.org/10.1186/s13071-017-2499-4

20. Biasizzo H, Šoba B, Ilovski F, Harlander M, Lukin M, Blatnik O, et al. Severe and rare case of human *Dirofilaria repens* infection with pleural and subcutaneous manifestations, Slovenia. Emerg Infect Dis. 2022;28:2504–7. PubMed https://doi.org/10.3201/eid2812.221366

21. Kotnik T, Rataj AV, Šoba B. *Dirofilaria repens* in dogs and humans in Slovenia. J Vet Res (Pulawy). 2022;66:117–23. PubMed https://doi.org/10.2478/jvetres-2022-0008

22. Xhekaj B, Kniha E, Wiesmüller L, Hoxha I, Eigner B, Stefanovska J, et al. Vector-borne pathogens in dogs from the Republic of Kosovo. Parasit Vectors. 2025;18:136. PubMed https://doi.org/10.1186/s13071-025-06777-0

23. Laynez-Roldán P, Martínez-de la Puente J, Montalvo T, Mas J, Muñoz J, Figuerola J, et al. Two cases of subcutaneous dirofilariasis in Barcelona, Spain. Parasitol Res. 2018;117:3679–81. PubMed https://doi.org/10.1007/s00436-018-6098-x

24. Raele DA, Pugliese N, La Bella G, Calvario A, Scarasciulli M, Vasco I, et al. Case report: molecular detection of *Dirofilaria repens* in an Italian patient after a stay in Tanzania. Am J Trop Med Hyg. 2021;104:2042–5. PubMed https://doi.org/10.4269/ajtmh.20-1360

25. Otranto D, Brianti E, Dantas-Torres F, Weigl S, Latrofa MS, Gaglio G, et al. Morphological and molecular data on the dermal microfilariae of a species of *Cercopithifilaria* from a dog in Sicily. Vet Parasitol. 2011;182:221–9. PubMed https://doi.org/10.1016/j.vetpar.2011.05.043

26. Pascucci I, Fico R, D'Angelo AR, Serini S, Camma C. First notification in Italy of cardiopulmonary filariosis (heartworm disease) in a wolf (*Canis lupus*). Vet Ital. 2007;43(4):843−50, 51−58.

27. Marcos R, Pereira C, Maia JP, Santos M, Luzzago C, Lauzi S, et al. The occurrence of the filarial nematode *Dirofilaria repens* in canine hosts from Maio Island, Cape Verde. J Helminthol. 2017;91:87–90. PubMed https://doi.org/10.1017/S0022149X16000067

28. Casiraghi M, Anderson TJ, Bandi C, Bazzocchi C, Genchi C. A phylogenetic analysis of filarial nematodes: comparison with the phylogeny of *Wolbachia* endosymbionts. Parasitology. 2001;122:93–103. PubMed https://doi.org/10.1017/S0031182000007149

29. Bozidis P, Sakkas H, Pertsalis A, Christodoulou A, Kalogeropoulos CD, Papadopoulou C. Molecular analysis of *Dirofilaria repens* isolates from eye-care patients in Greece. Acta Parasitol. 2021;66:271–6. PubMed https://doi.org/10.1007/s11686-020-00257-4

30. Ugolini S, Lima M, Maffi M, Pierangeli F, Vastano M, Gargano T, et al. *Dirofilaria repens* testicular infection in child, Italy. Emerg Infect Dis. 2022;28:2569–72. PubMed https://doi.org/10.3201/eid2812.220424

31. Fagundes-Moreira R, Bezerra-Santos MA, May-Junior JA, Berger L, Baggio-Souza V, Souza UA, et al. *Dirofilaria immitis* and *Onchocercidae* spp. in wild felids from Brazil. Parasitol Res. 2024;123:195. PubMed https://doi.org/10.1007/s00436-024-08209-x

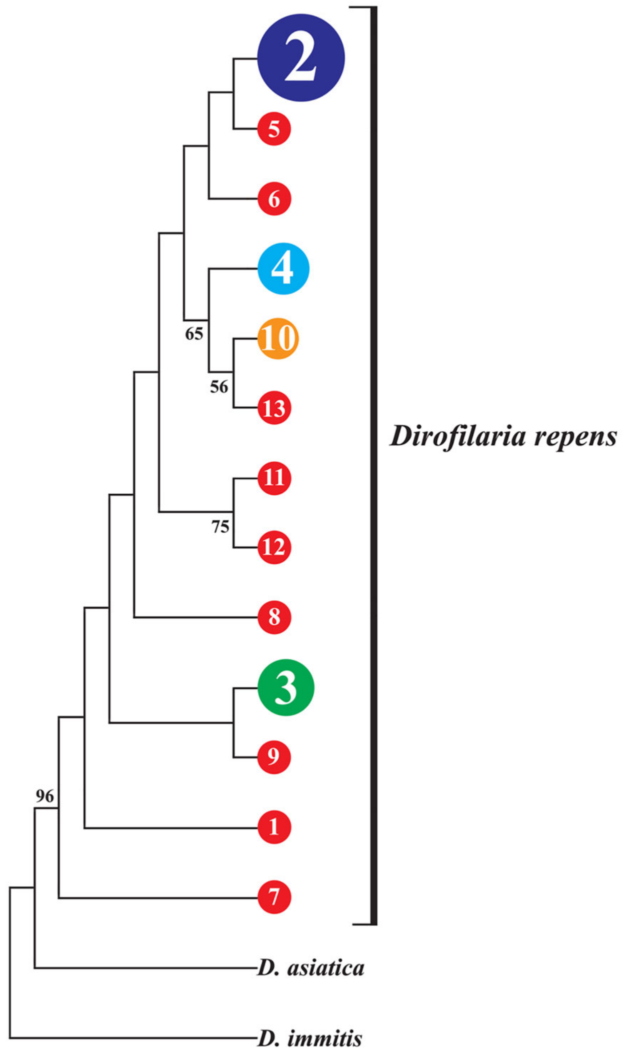

Appendix Figure. Neighbor-joining tree of *Dirofilaria repens* haplotypes (n = 13), based on 570 bp sequences of the mitochondrial DNA *cox1* gene (*D. asiatica* and *D. immitis* are outgroup taxa). The number in circles is the number of a haplotype (Appendix Table 2). The isolate from Estonia is haplotype 1. Haplotypes represented by a unique sequence are in red circles, whereas haplotypes represented by two or more sequences are in different colors (Appendix Table 2). Bootstrap values >50% are indicated at the branches.
